# B7-H3 promotes aerobic glycolysis and chemoresistance in colorectal cancer cells by regulating HK2

**DOI:** 10.1038/s41419-019-1549-6

**Published:** 2019-04-05

**Authors:** Tongguo Shi, Yanchao Ma, Lei Cao, Shenghua Zhan, Yunyun Xu, Fengqing Fu, Cuiping Liu, Guangbo Zhang, Zhenxin Wang, Ruoqin Wang, Huimin Lu, Binfeng Lu, Weichang Chen, Xueguang Zhang

**Affiliations:** 1 Jiangsu Institute of Clinical Immunology, The First Affiliated Hospital of Soochow University, 708 Renmin Road, Suzhou, China; 2 Jiangsu Key Laboratory of Clinical Immunology, Soochow University, 708 Renmin Road, Suzhou, China; 3 Jiangsu Key Laboratory of Gastrointestinal tumor Immunology, The First Affiliated Hospital of Soochow University, 708 Renmin Road, Suzhou, China; 4 Department of Gastroenterology, The First Affiliated Hospital of Soochow University, 188 Shizi Road, Suzhou, China; 5 Institute of Paediatric Research, Affiliated Children’s Hospital of Soochow University, 92 Zhongnan Road, Suzhou, China; 6 Department of Immunology, School of Medicine, University of Pittsburgh, EBST E1047, 200 Lothrop Street, Pittsburgh, PA USA

## Abstract

Accumulating evidence suggests that aerobic glycolysis is important for colorectal cancer (CRC) development. However, the underlying mechanisms have yet to be elucidated. B7-H3, an immunoregulatory protein, is broadly overexpressed by multiple tumor types and plays a vital role in tumor progression. In this study, we found that overexpression of B7-H3 effectively increased the rate of glucose consumption and lactate production, whereas knockdown of B7-H3 had the opposite effect. Furthermore, we showed that B7-H3 increased glucose consumption and lactate production by promoting hexokinase 2 (HK2) expression in CRC cells, and we also found that HK2 was a key mediator of B7-H3-induced CRC chemoresistance. Depletion of HK2 expression or treating cells with HK2 inhibitors could reverse the B7-H3-induced increase in aerobic glycolysis and B7-H3-endowed chemoresistance of cancer cells. Moreover, we verified a positive correlation between the expression of B7-H3 and HK2 in tumor tissues of CRC patients. Collectively, our findings suggest that B7-H3 may be a novel regulator of glucose metabolism and chemoresistance via controlling HK2 expression in CRC cells, a result that could help develop B7-H3 as a promising therapeutic target for CRC treatment.

## Introduction

Colorectal cancer (CRC) is the third most prevalent cancer type in the world^1^. Although screening and radical surgical resection have significantly improved the 5-year survival rate of patients with CRC in the early stage, the majority of patients are diagnosed at advanced stages^2^. Unfortunately, very few therapy options are currently available for effective treatment of advanced CRC. Therefore, it is imperative to understand the molecular mechanisms underlying CRC progression and to identify precise and effective biomarkers.

B7-H3, also known as CD276, is an important immune checkpoint member of the B7-CD28 family^3^. As a type I transmembrane protein, two B7-H3 isoforms (4IgB7-H3 and 2Ig-B7-H3) have been identified; 4Ig-B7-H3 is the main isoform in humans and 2Ig-B7-H3 is the only isoform in mice^4^. Because of the lack of an identified receptor, the immunologic function of B7-H3 remains controversial, with conflicting costimulatory and coinhibitory functions^5^. However, B7-H3 has been reported to be a pivotal non-immunologically multifunctional protein involved in the regulation of many key cellular events. Interestingly, accumulated evidence indicates that aberrant expression of B7-H3 is a common characteristic of CRC and is consistently correlated with poor patient prognosis, suggesting its emerging importance in CRC progression^6,7^. A previous report showed that B7-H3 could promote epithelial to mesenchymal transition (EMT) in CRC cells, evidenced by decreasing the expression of E-cadherin and β-catenin and up-regulating N-cadherin and vimentin expression^8^. In addition, the administration of anti-B7-H3-drug conjugates to various human CRC xenografts could simultaneously destroy both tumor cells and tumor vasculature^9^. Moreover, B7-H3 could upregulate BRCA1/BRCA2-containing complex subunit 3 (BRCC3) or X-ray repair cross complementing group 1 (XRCC1) expression to antagonize DNA damage caused by 5-fluorouracil (5-FU) or oxaliplatin (L-OHP)^10,11^. Although those studies have suggested multiple roles for B7-H3 in CRC, it is necessary to understand the exact roles of B7-H3 in the development and progression of CRC.

Cancer cell metabolism is characterized by an increase in glycolysis and lactate production even in the presence of abundant oxygen, known as the Warburg effect or aerobic glycolysis^12^. Aerobic glycolysis confers on cancer cells a growth advantage by providing energy and biosynthesis building blocks^13^. It has been widely accepted that aerobic glycolysis is a distinctive hallmark of cancer, and antitumor therapeutic agents targeting aerobic glycolysis are being developed^14^. Accumulated evidence has indicated that oncogenic alterations may participate in the regulation of aerobic glycolysis^15^. For instance, hypoxia-inducible factor 1α (HIF-1α), which is rapidly upregulated under hypoxic conditions, increases the expression of glycolysis-associated proteins, such as glucose transporters and glycolytic enzymes^16^. Another important oncogenic protein, c-MYC, was reported to promote glycolysis through transactivating the glycolytic enzyme genes^17^. Oncogenic signaling pathways, such as the PI3K/Akt、STAT3 and Wnt/β-catenin pathways, are also known as regulators of cancer cell metabolism^18–20^. In addition, miRNA-mediated posttranscriptional regulation is involved in regulating cancer aerobic glycolysis^21^. These studies suggest that aerobic glycolysis in cancer cells is far more complicated than expected and warrants further investigation.

Despite a promoting aerobic glycolysis of B7-H3 in breast cancer, evidenced by increasing glucose uptake and lactate production^22,23^, the effects of B7-H3 on aerobic glycolysis in CRC remain largely unknown. In this study, we investigated whether and how B7-H3 modulated glucose metabolism in CRC. We showed here that B7-H3 enhanced aerobic glycolysis by upregulating the expression of the glycolytic enzyme hexokinase 2 (HK2), a key mediator of aerobic glycolysis, in CRC cells. B7-H3 promoted HK2 expression by activating STAT3 signaling. Importantly, we demonstrated that HK2 expression was critical for B7-H3-mediated CRC chemoresistence both in vitro and in vivo. These findings revealed a previously unrecognized mechanism of B7-H3 in human CRC by affecting the aerobic glycolysis and chemoresistence through regulation of HK2.

## Results

### B7-H3 promotes glucose consumption and lactate production in colorectal cancer cells

**Fig. 1:**
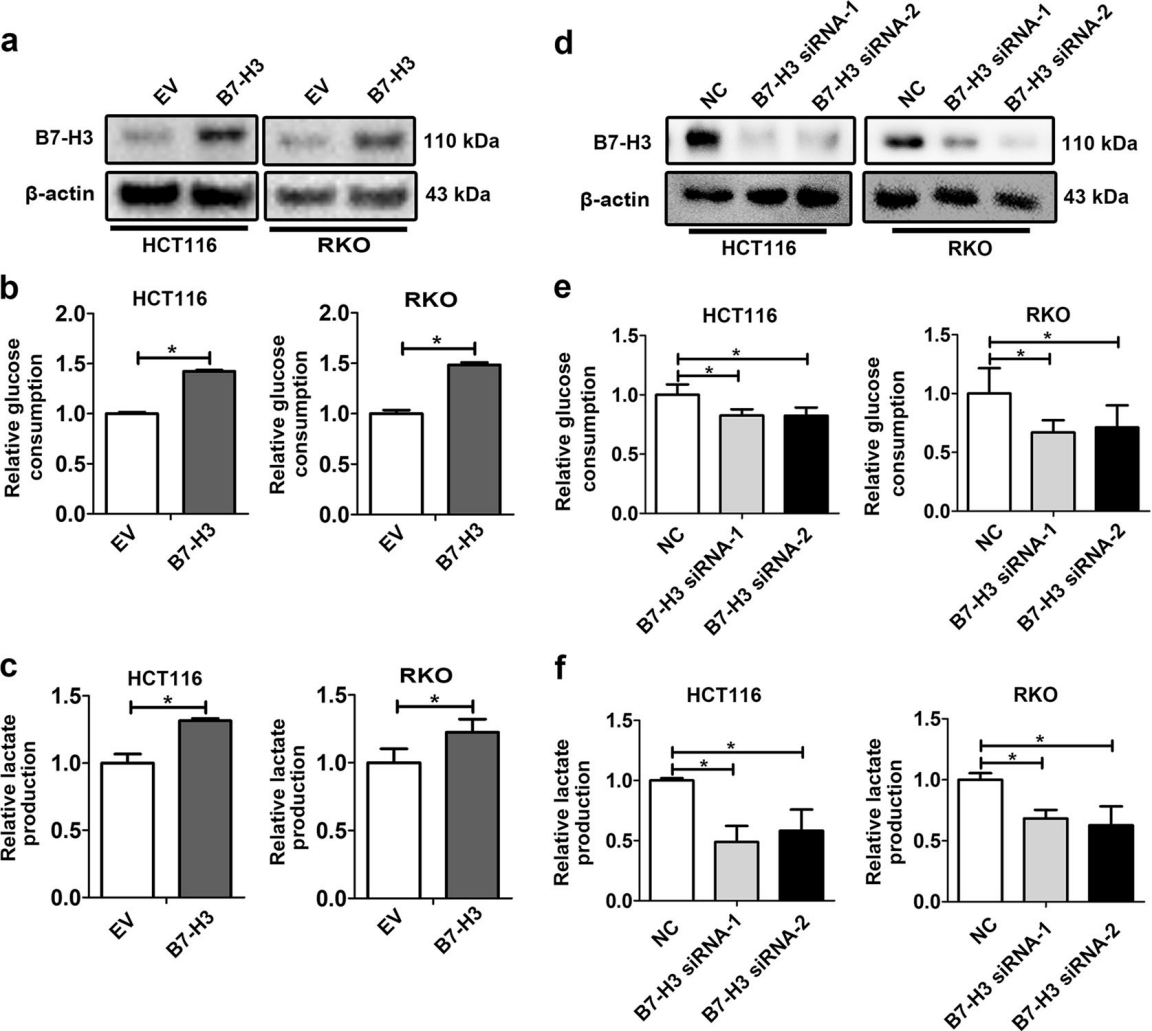
B7-H3 promoted aerobic glycolysis in CRC cells. **a** B7-H3 protein levels in both B7-H3-overexpressing HCT116 and RKO cells were analyzed by western blot. β-actin served as a loading control. **b**, **c** Glucose consumption (**b**) and lactate production (**c**) were measured in both B7-H3-overexpressing HCT116 and RKO cells. **d** B7-H3 protein levels in both HCT116 and RKO cells were analyzed by western blot after transfection with siRNA negative control (NC), B7-H3 siRNA-1 or B7-H3 siRNA-2. β-actin served as a loading control. **e**, **f** Glucose consumption (**e**) and lactate production (**f**) were measured in both HCT116 and RKO cells transfected with NC, B7-H3 siRNA-1 or B7-H3 siRNA-2. Values were expressed as means (SEMs). Five samples were analyzed per condition, and the experiments were performed in triplicate. *P < 0.05

To determine the effects of B7-H3 on glucose consumption and lactate production in colorectal cancer cells, we established two CRC cell lines stably expressing B7-H3, with B7-H3 protein levels more than twice as high as that of control cells (Fig. 1a). Both glucose consumption and lactate production were significantly increased in B7-H3-overexpression HCT116 and RKO cells compared with control cells without B7-H3 overexpression (Fig. 1b, c). On the other hand, we knocked down the expression of B7-H3 in HCT116 and RKO cells using two independent siRNAs specific for B7-H3 (B7-H3 siRNA-1 and B7-H3 siRNA-2), which reduced the expression of B7-H3 to less than 70% of that of the scrambled control (Fig. 1d). Reduction of B7-H3 significantly decreased glucose consumption and lactate production in both cell lines (Fig. 1e, f). Collectively, these data demonstrate that B7-H3 promoted aerobic glycolysis and lactate production in CRC cells.

### B7-H3 controls expression of HK2 via STAT3

**Fig. 2:**
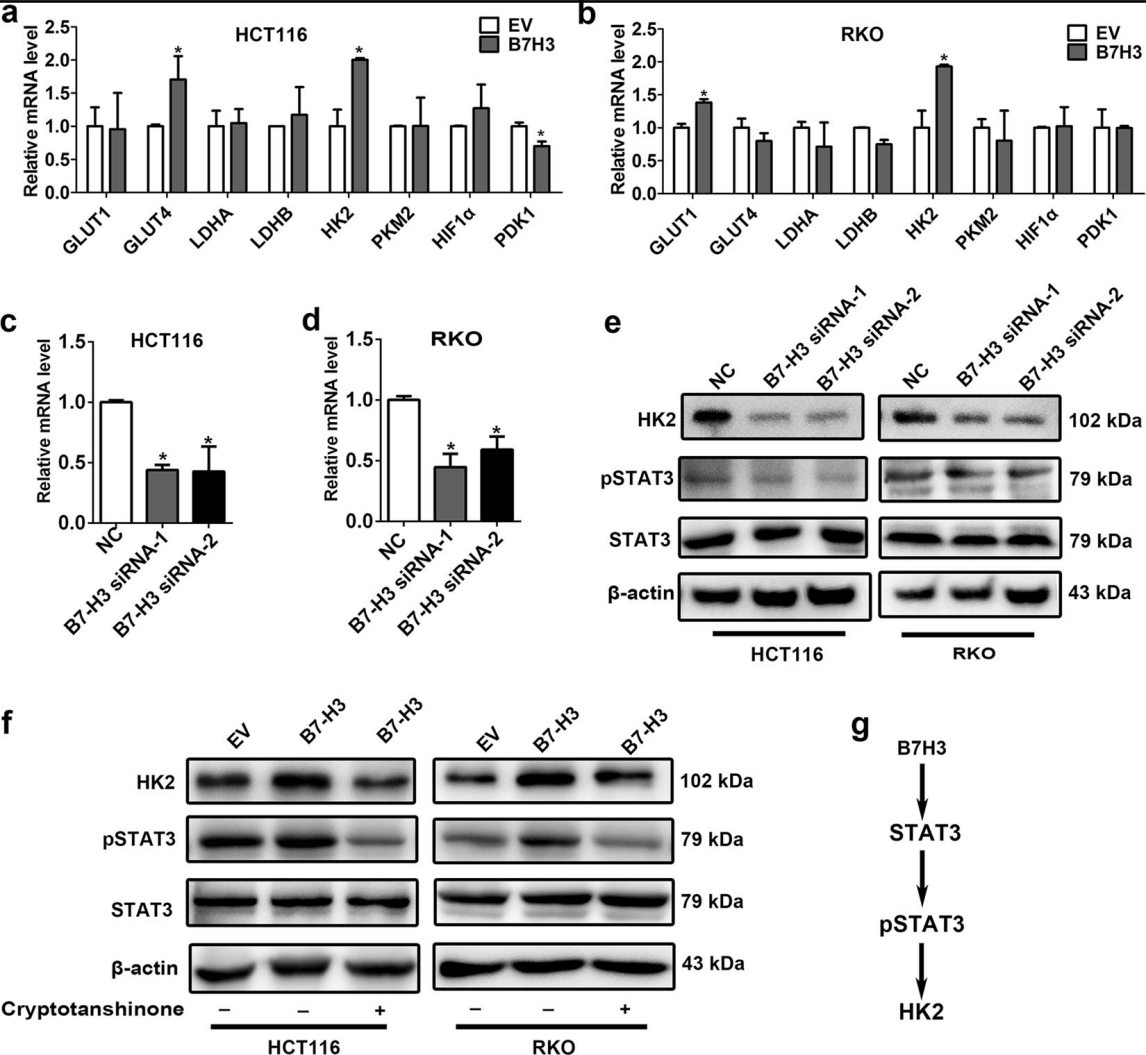
B7-H3 promoted the expression of HK2 in CRC cells. **a**, **b** The expression of glycolysis-related genes was detected by RT-qPCR in both B7-H3-overexpressing HCT116 (**a**) and RKO (**b**) cells. **c**, **d** The mRNA level of HK2 was detected by RT-qPCR in both HCT116 (**c**) and RKO (**d**) cells after transfection with siRNA NC, B7-H3 siRNA-1 or B7-H3 siRNA-2. **e** HK2 protein level and STAT3 activation (examined by the p-STAT3 expression level) were detected by western blot in both HCT116 and RKO cells after transfection with NC, B7-H3 siRNA-1 or B7-H3 siRNA-2. β-actin served as a loading control. **f** HK2 protein level and STAT3 activation (examined by the p-STAT3 expression level) were detected by western blot in both B7-H3-overexpressing HCT116 and RKO cells. β-actin served as a loading control. **g** Schematic representation of the proposed B7-H3/STAT3/HK2 axis. Values are expressed as means (SEMs). Five samples were analyzed per condition, and the experiments were performed in triplicate. *P < 0.05

To investigate how B7-H3 regulates aerobic glycolysis in CRC, we analyzed the expression of a spectrum of key glycolysis-related genes, including Glucose transporter 1 (GLUT1), Glucose transporter 4 (GLUT4), Lactate dehydrogenase A (LDHA), Lactate dehydrogenase B (LDHB), HK2, pyruvate kinase M 2 (PKM2), HIF-1α and pyruvate dehydrogenase kinase 1 (PDK1) in B7-H3-overexpressing HCT116, and RKO cells with real-time quantitative PCR (RT-qPCR) (Fig. 2a, b). We found that the expression level of HK2, a known mediator of aerobic glycolysis, was the most increased in both HCT116 and RKO cells with overexpression of B7-H3 (Fig. 2a, b). In contrast, knockdown of B7-H3 significantly reduced the mRNA and protein levels of HK2 in HCT116 and RKO cells (Fig. 2c, d). In addition, western blot analysis revealed that knockdown of B7-H3 significantly reduced the protein levels of HK2 in HCT116 and RKO cells (Fig. 2e), while overexpression of B7-H3 resulted in a significant increase in HK2 protein expression (Fig. 2f). Thus, our results suggest that B7-H3 positively regulated HK2 expression.

We next sought to explore the signaling pathways by which B7-H3 mediated the expression of HK2. Previous studies have shown that the expression of HK2 could be regulated by STAT3 in tumor cells such as MCF7, HepG2, and A549 cells^24,25^. In addition, STAT3 signaling is known to be a downstream target of B7-H3 in CRC^26,27^. We hypothesized that the upregulation of HK2 in both B7-H3-overexpressing CRC cells might be STAT3 dependent. Indeed, B7-H3 siRNA treatment reduced the activity of STAT3 (Fig. 2e). Conversely, increased STAT3 activity was observed in both B7-H3-overexpressing HCT116 and RKO cells (Fig. 2f). Moreover, we observed that cryptotanshinone, a STAT3 phosphorylation inhibitor, that blocked the phosphorylation of STAT3, significantly decreased the expression of HK2 in both B7-H3-overexpressing HCT116 and RKO cells (Fig. 2f). These results suggest that B7-H3 could regulate the expression of HK2 by the STAT3 signaling pathway in CRC cells (Fig. 2g).

### B7-H3 promotes aerobic glycolysis via HK2

**Fig. 3:**
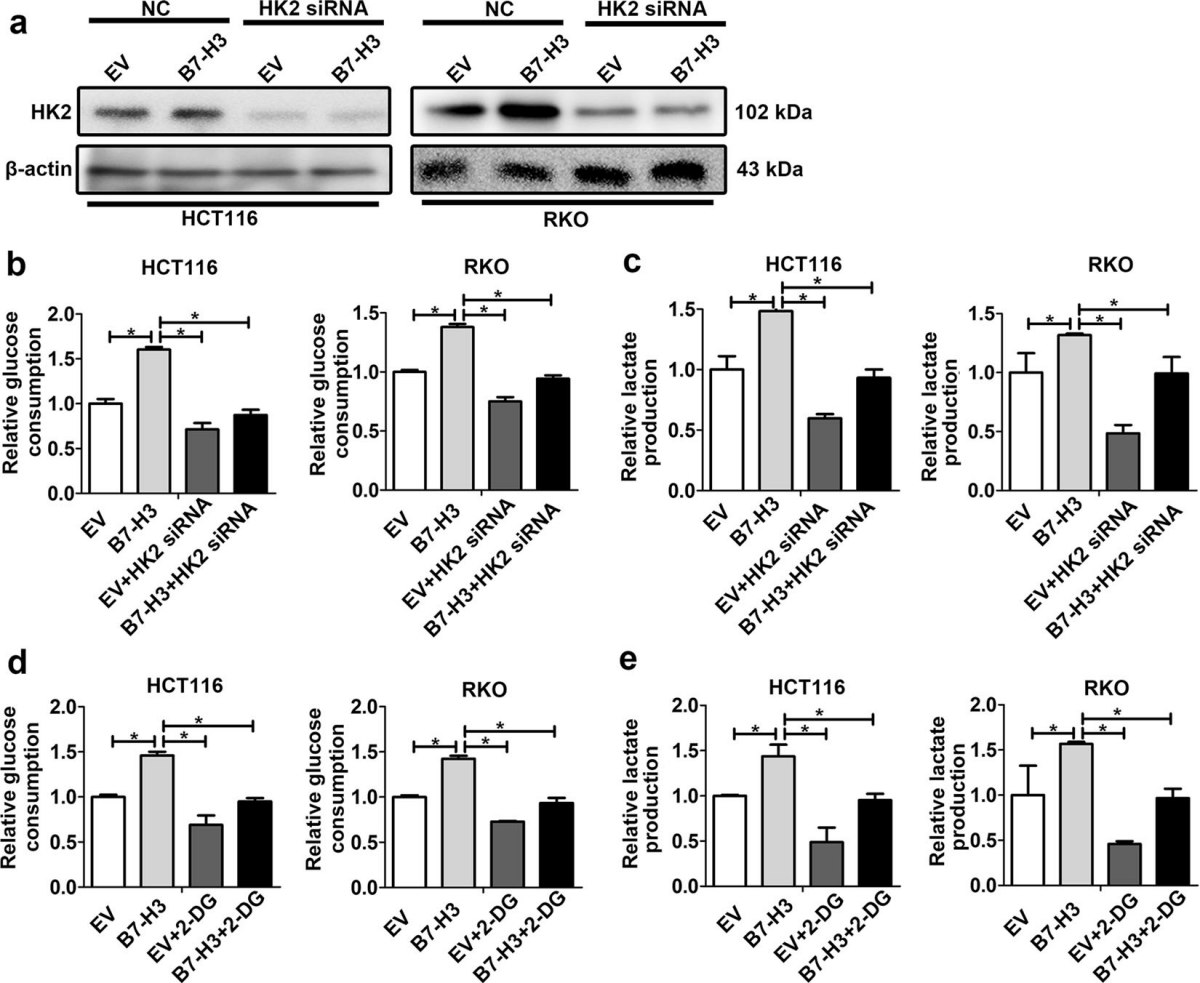
B7-H3 regulated glycolysis through HK2. **a** The protein level of HK2 in B7-H3-overexpressing HCT116 or RKO cells after transfection with siRNA negative control (NC) or HK2 siRNA transfection were analyzed by western blot. β-actin served as a loading control. **b**, **c** Glucose consumption (**b**) and lactate production (**c**) were measured in B7-H3-overexpressing HCT116 or RKO cells transfected with NC or HK2 siRNA. **d**, **e** Glucose consumption (**d**) and lactate production (**e**) were measured in B7-H3-overexpressing HCT116 or RKO cells treated with PBS or 2-DG. Values are expressed as means (SEMs). Five samples were analyzed per condition, and the experiments were performed in triplicate. *P < 0.05

When HK2 was downregulated by HK2 siRNA, B7-H3-induced increase in glucose uptake and lactate production was obviously attenuated (Fig. 3a–c). Additionally, 2-Deoxy-D-glucose (2-DG), a HK2 inhibitor, was used to treat B7-H3-overexpressing HCT116 and RKO cells. Treatment with 2-DG abolished the B7-H3-induced increase in glucose uptake and lactate production (Fig. 3d, e). These results indicate that B7-H3-induced aerobic glycolysis was HK2 dependent.

### B7-H3 increases CRC cell chemoresistance via HK2 in vitro

In addition to promoting tumorigenesis, aerobic glycolysis of cancer cells provides an environment that often increases drug resistance^28^. It has been shown that knockdown of HK2 can sensitize glioblastoma multiforme cells to therapeutic drugs^29^. Here, we chose 25 nM HK2 siRNA or 5 mM 2-DG, a concentration resulting in around 10% cells viablility in both HCT116 and RKO cells, to evaluate whether HK2 was critical for B7-H3-regulated cancer chemoresistance.

**Fig. 4:**
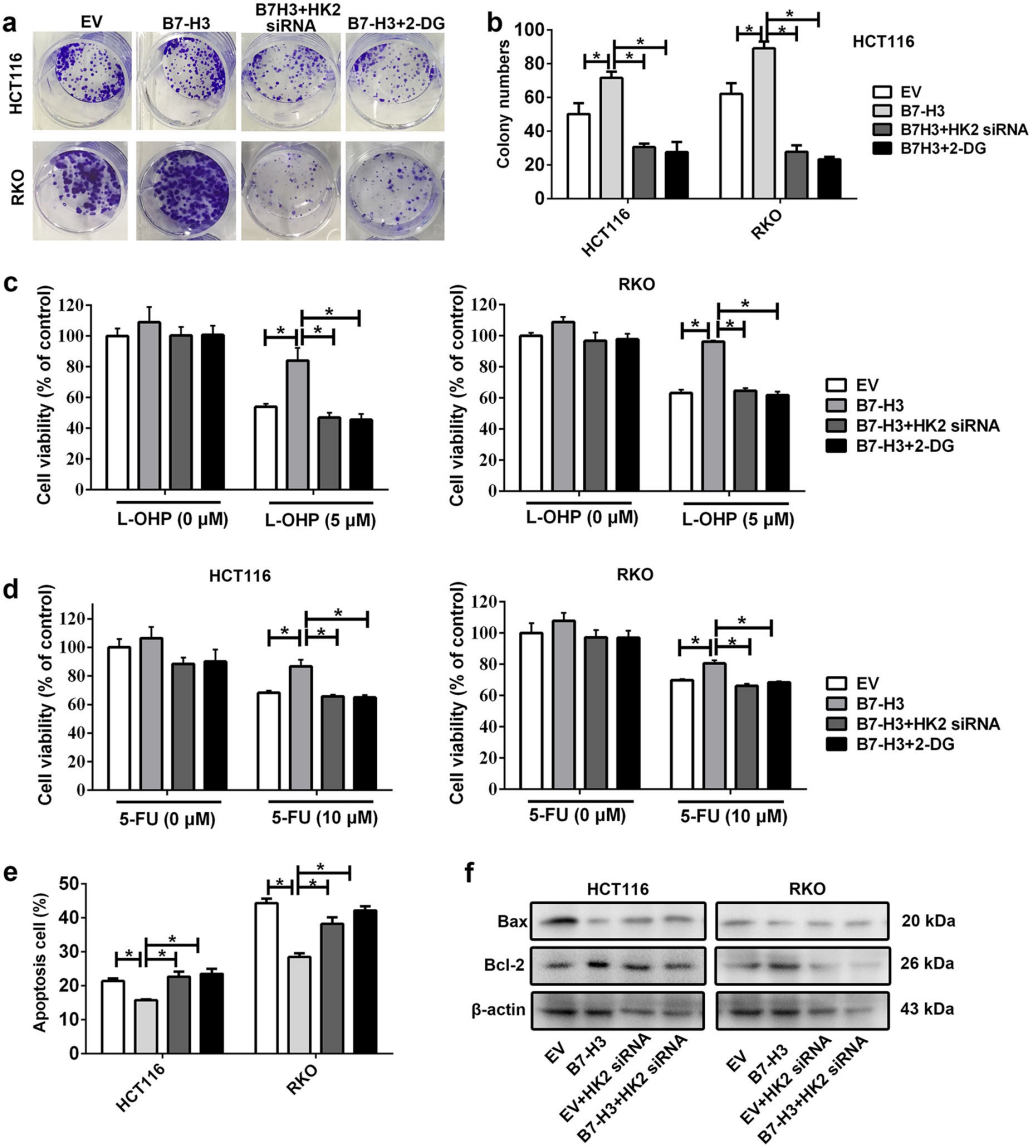
The effect of B7-H3 on the chemoresistance of CRC cells was HK2 dependent in vitro. **a**, **b** Colony formation assay of B7-H3-overexpressing HCT116 or RKO cells exposed to L-OHP. The chemoresistance of B7-H3 was abolished by HK2 siRNA or 2-DG. **c**, **d** Cell viability was analyzed using the CCK-8 kit. B7-H3-overexpressing HCT116 or RKO cells showed resistance to L-OHP (**c**) or 5-FU (**d**). The chemoresistance of B7-H3 was abolished by HK2 siRNA or 2-DG. **e** B7-H3-overexpressing HCT116 or RKO cells showed less cell apoptosis than control cells (EV) after L-OHP treatment. The effect of B7-H3 on cell apoptosis was abolished by HK2 siRNA or 2-DG. **f** The protein levels of Bcl-2 and Bax were detected by western blot in both B7-H3-overexpressing HCT116 and RKO cells. The effect of B7-H3 on Bcl-2 and Bax expression was abolished by HK2 siRNA. β-actin served as a loading control. Values are expressed as means (SEMs). Five samples were analyzed per condition, and the experiments were performed in triplicate. *P < 0.05

The effect of B7-H3 on CRC cell chemoresistance was determined with clonogenic assay. As shown in Fig. 4a, b, B7-H3 overexpression significantly enhanced the clonogenic potential of HCT116 and RKO cells upon exposure to L-OHP. In addition, cell viability analysis showed that B7-H3 enhanced L-OHP resistance of HCT116 and RKO cells (Fig. 4c). These results were further confirmed by treatment with another chemotherapy drug, 5-FU (Fig. 4d). Furthermore, flow cytometry analysis showed less apoptosis in B7-H3-overexpressing HCT116 and RKO cells than in control cells treated with L-OHP (Fig. 4e and Supplemental Fig. S1). Finally, enhanced B cell lymphoma 2 (Bcl-2) expression and reduced Bcl-2-Associated X (Bax) expression was observed in B7-H3-overexpressing HCT116 and RKO cells that were treated with L-OHP (Fig. 4f).

To examine whether the CRC cell resistance to chemotherapeutic drugs by B7-H3 overexpression occurs through elevated glucose metabolism, HK2 siRNA or 2-DG was used to treat B7-H3-overexpressing HCT116 and RKO cells. We found that chemoresistance of B7-H3-overexpressing HCT116 and RKO cells to L-OHP and 5-FU was reversed by HK2 siRNA or 2-DG (Fig. 4).

### B7-H3/HK2 pathway induces CRC chemoresistance in vivo

**Fig. 5:**
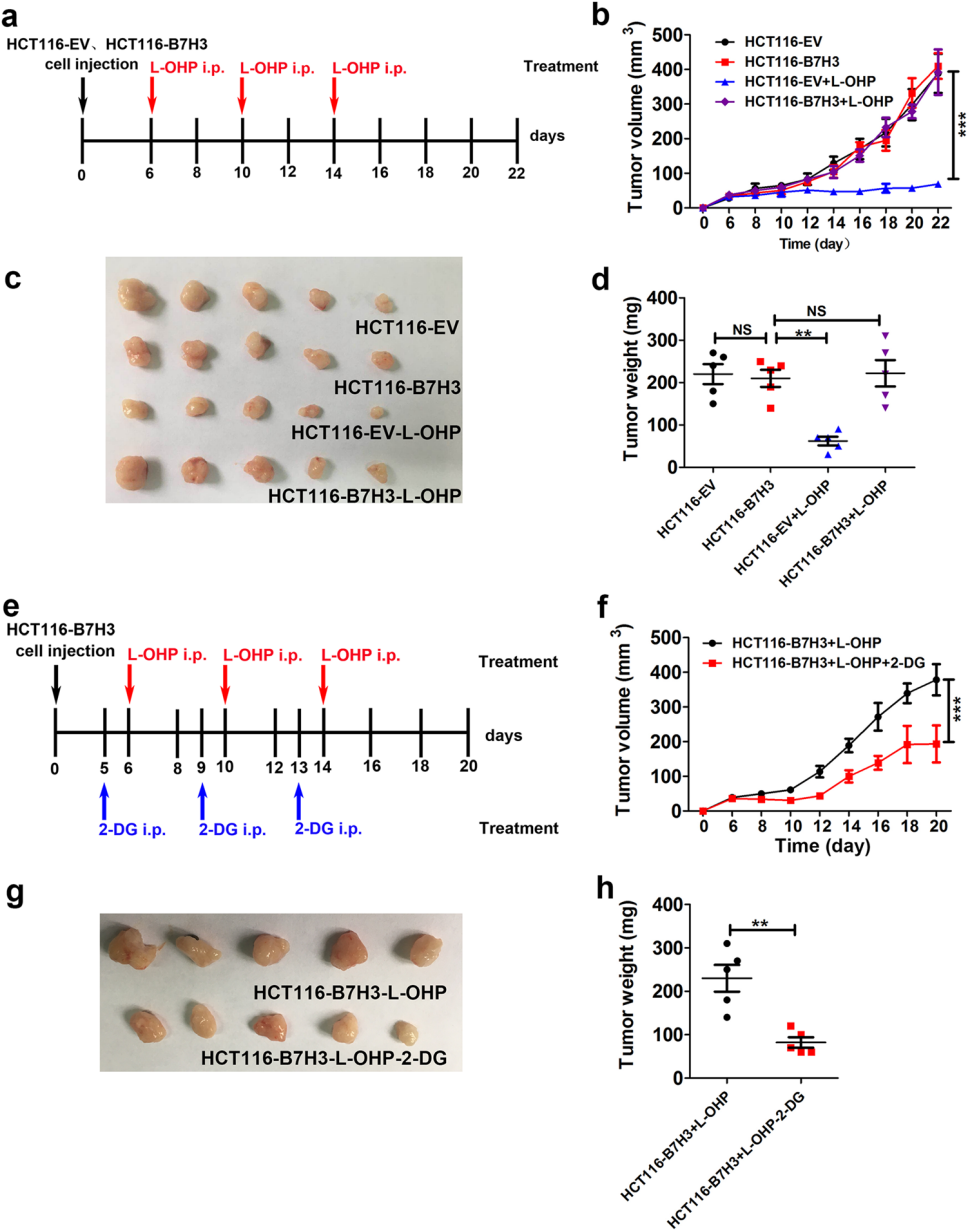
The effect of B7-H3 on the chemoresistance of CRC cells was HK2 dependent in vivo. **a** Schematic overview of the xenograft tumor model and L-OHP treatment. **b** The volumes of the B7-H3-overexpressing HCT116 tumors in nude mice following L-OHP treatment. **c** A representative image of B7-H3-overexpressing HCT116 tumors in nude mice following L-OHP treatment. **d** The weights of the B7-H3-overexpressing HCT116 tumors in nude mice following L-OHP treatment. **e** Schematic overview of the xenograft tumor model and 2-DG or L-OHP treatment. **f** The volumes of the B7-H3-overexpressing HCT116 tumors in nude mice following 2-DG and L-OHP treatment. **g** A representative image of the overexpressing B7-H3 HCT116 tumors in nude mice following 2-DG and L-OHP treatment. **h** The weights of the B7-H3-overexpressing HCT116 tumors in nude mice following 2-DG and L-OHP treatment. Values are expressed as means (SEMs). *n* = 5 mice per group. *P < 0.05

To confirm the chemoresistance effect of B7-H3 on CRC in vivo, xenograft models of B7-H3-overexpressing HCT116 tumors or control tumors in nude mice were established, and L-OHP was administered at different timepoints (Fig. 5a). The tumor sizes, images and mass revealed that B7-H3 had no effect on untreated HCT116 tumor growth (Fig. 5b–d). However, tumors that were derived from B7-H3-overexpressing HCT116 cells were significantly more resistant to L-OHP than did tumors derived from control vector HCT116 cells (Fig. 5b–d). We next confirmed whether B7-H3-regulated CRC chemoresistance was HK2 dependent in vivo. L-OHP and 2-DG were administered to the mice injected with B7-H3-overexpressing HCT116 cells at different timepoints (Fig. 5e). We found that 2-DG significantly reversed the resistance of B7-H3-overexpressing HCT116 tumors to L-OHP in vivo (Fig. 5f–h). The data suggested that B7-H3-induced glycolysis promoted CRC cell chemoresistance, and this was reversed by glycolysis inhibition with 2-DG treatment in vivo.

### B7-H3 protein levels positively correlate with HK2 expression in CRC patient tumor tissue specimens

**Fig. 6:**
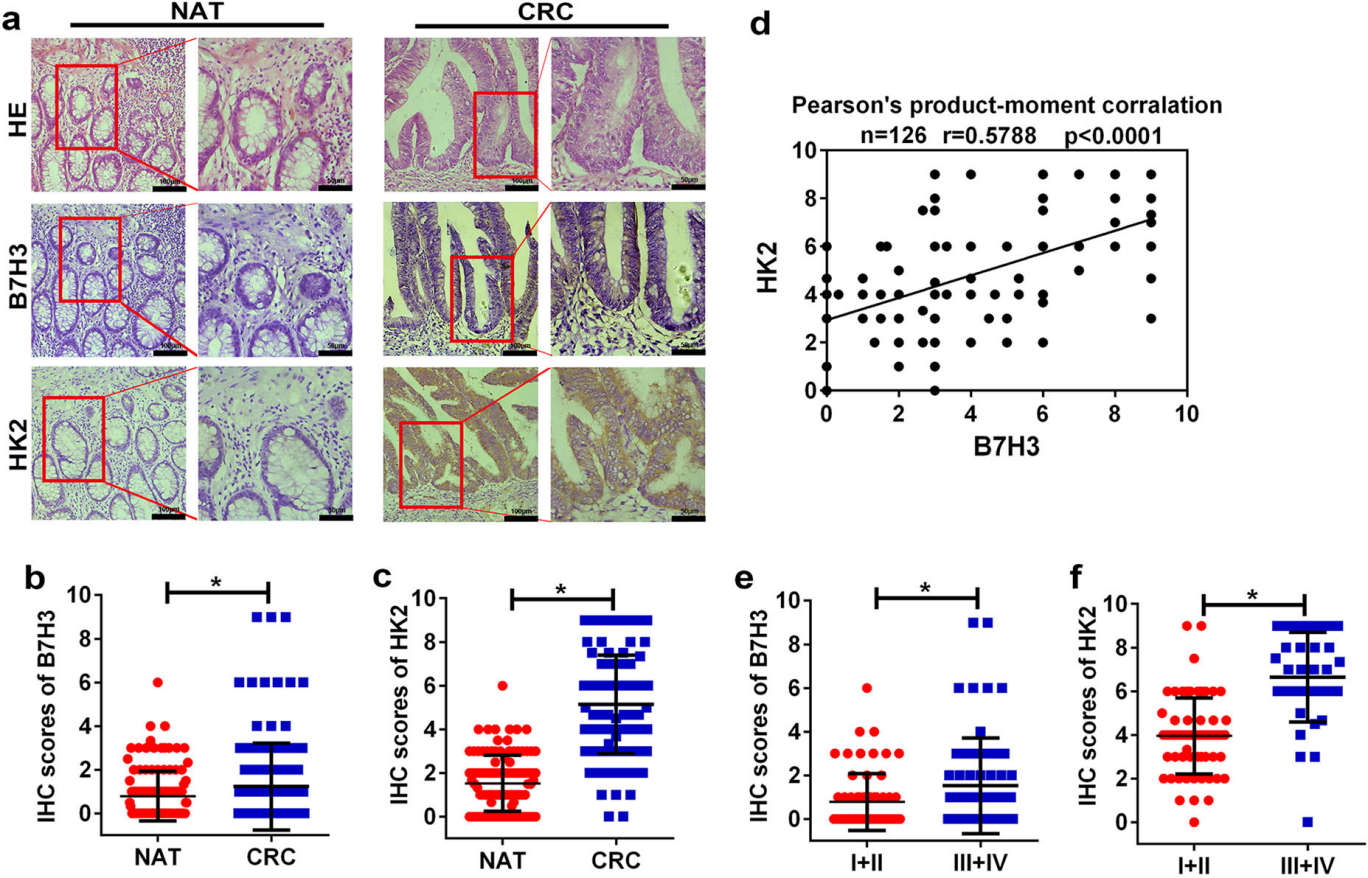
HK2 protein levels positively correlated with B7-H3 expression in CRC patient specimens. **a** Images of IHC analysis of B7-H3 and HK2 protein expression and hematoxylin and eosin (H&E) staining of CRC (*n* = 126) tissue sections. One representative image is shown. **b**, **c** B7-H3 (**b**) and HK2 (**c**) protein expression based on their staining index in nonmalignant adjacent tissues (NAT) and CRC specimens. **d** Correlation analysis of the staining index of expression levels of B7-H3 and HK2 protein in human CRC specimens (*n* = 126). **e**, **f** B7-H3 (**e**) and HK2 (**f**) protein expression based on their staining index in CRC specimens at different clinical stages. Values are expressed as means (SEMs). *P < 0.05

Previous studies suggested that B7-H3 was aberrantly expressed in colorectal cancer and consistently correlated with poor colon cancer patient outcomes^8,30^. To investigate the clinical correlation between B7-H3 and HK2 protein levels in CRC patient specimens, we analyzed 126 pairs of the primary tumor lesions and corresponding normal adjacent tissues in Chinese patients with CRC. IHC analysis showed that both B7-H3 and HK2 proteins were significantly upregulated in CRC samples compared with normal adjacent tissues (Fig. 6a–c). Moreover, HK2 was positively correlated with B7-H3 expression in this cohort (Fig. 6d). Additionally, both B7-H3 and HK2 expression increased with tumor stage. The levels of B7-H3 and HK2 were higher in advanced clinical stages (III and IV) than in early stages (I and II) (Fig. 6e, f). Thus, the levels of B7-H3 and HK2 were positively correlated in human CRC specimens.

## Discussion

In this study, we focused our research on the regulation of CRC aerobic glycolysis and chemoresistance by B7-H3. Although it has been reported that B7-H3 can regulate aerobic glycolysis in breast cancer, the underlying molecular mechanism of B7-H3 in the regulation of aerobic glycolysis in CRC remains unknown^22,23^. Here, we showed that B7-H3 promoted glucose uptake and lactate production in CRC cells. Considering the aberrant expression of B7-H3 in CRC^6,7^ (Fig. 6a) and the emerging importance of aerobic glycolysis for cancer development, we therefore conclude that B7-H3 may play a critical role in the regulation of glucose metabolism in CRC cells.

Previous studies showed that HIF-1α played key roles in regulating aerobic glycolysis under hypoxic conditions^16,31^. It has also been shown that B7-H3 could regulate aerobic glycolysis via HIF-1α in breast cancer cells^22^. However, we observed that the protein level of HIF-1α was only marginally affected by B7-H3 (Supplemental Fig. S2), suggesting exclusion of HIF-1α involvement. Moreover, given that the basal protein level of HIF-1α is very low under normoxia, it is inexplicable why cancer cells adapt to glycolysis, which is less efficient, for energy sources even in the presence of a rich oxygen supply^21^. In addition, Nunesxavier et al. reported that B7-H3 overexpression did not alter activation of the Akt/mTOR pathway but that the high B7-H3 expression levels in tumor cells increased cell survival and glycolysis^23^. Therefore, we surmised that other mechanisms might be involved in B7-H3-mediated aerobic glycolysis in CRC cells. Indeed, we found that the mRNA and protein levels of HK2 in CRC cells could be modulated by B7-H3 (Fig. 2).

Until now, hexokinases (HKs) are tissue-specific isoenzymes that catalyze the first step of glucose metabolism by phosphorylating glucose to glucose-6-phosphatase^32^. The mammalian HK family has four isoforms, HK1, 2, 3, and 4. Among these isoforms, HK2 is critically important for aerobic glycolysis in multiple cancer types, including glioblastoma multiforme, breast and ovarian cancer^29,33,34^. Overexpression of HK2 is also observed in multiple tumor tissues and is associated with poor prognosis in tumor patients^35^. 2-DG, a well-known HK2 inhibitor, induces cancer cell death by depleting intracellular glucose levels^36^. Therefore, HK2 is regarded as a key player in aerobic glycolysis and has been proposed as a therapeutic target for cancers^37^. The expression of HK2 in cancer cells could be regulated at the transcriptional and post-transcriptional level. Previous studies showed that HK2 was directly regulated by miR-199a-5p in liver cancer^38^; by miR-155 in lung cancer^39^; and by miR-143 in colon cancers^40^. Zhuo et al. revealed that PI3K/Akt signaling inhibited cell apoptosis and promoted tumor growth by upregulating HK2 expression in pediatric osteosarcoma^41^. Additionally, STAT3 signaling has been shown to accelerate aerobic glycolysis by upregulating HK2 in bladder, breast and hepatocellular carcinoma^24,42,43^. In this study, we found that B7-H3 could activate STAT3 and then upregulate HK2 expression in CRC cells (Fig. 2e, f). Importantly, cryptotanshinone could inhibit the expression of HK2 induction by B7-H3 in CRC cells (Fig. 2f). These results suggested that B7-H3 could regulate the expression of HK2 via the STAT3 signaling pathway (Fig. 2g).

Mounting evidence suggests that B7-H3 is intimately associated with chemotherapeutic resistance. For instance, B7-H3 silencing increased the sensitivity of Maver and Z138 cells to rituximab and bendamustine and enhanced drug-induced apoptosis in mantle cell lymphoma^44^. ShRNA targeting B7-H3 significantly enhanced the sensitivity of U937 cells to first-line chemotherapy drugs (idarubicin and cytarabine) used in acute monocytic leukemia^45^. Upregulation of B7-H3 diminished the sensitization role of astragaloside IV, a component of Traditional Chinese Medicine *Astragalus membranaceus*, in cellular responses to cisplatin in non-small cell lung cancer^46^. Moreover, overexpression of B7-H3 could affect the efficacy of DNA damage-inducing anticancer drugs in CRC^10,11^. Herein, we showed novel relationships between B7-H3-mediated aerobic glycolysis and chemoresistance. Previous studies have demonstrated that the expression levels of HK2, PKM2, LDHA, and GLUT1 are correlated with the development of chemoresistance in malignant tumors^47^. Glycolytic inhibitors, such as 3-bromopyruvate and 2-DG, could enhance the sensitivity of tumor cells to chemotherapy drugs^48,49^. In the present study, we found that inhibition of aerobic glycolysis by 2-DG or knockdown of HK2 expression effectively abolished B7-H3 overexpression-induced resistance to L-OHP and 5-FU in vitro and in vivo (Figs. 4 and 5). Our findings revealed that B7-H3-mediated aerobic glycolysis played a critical role in the development of chemoresistance in CRC cells and suggested that B7-H3 might be a potential target to prevent the development of CRC chemoresistance.

In sum, we investigated the function of B7-H3 in CRC aerobic glycolysis and elucidated an important molecular mechanism of B7-H3 in the development of CRC chemoresistance. We revealed that B7-H3 mediated the activation of STAT3 and subsequent expression of HK2, to promote glycolysis in CRC cells. Moreover, B7-H3-mediated aerobic glycolysis could enhance the chemoresistance of CRC cells in vitro and in vivo. Therefore, our findings have uncovered that B7-H3 is a novel regulatory factor of glucose metabolism and chemoresistance via controlling HK2 expression in CRC cells, suggesting B7-H3 as a promising therapeutic target for CRC treatment.

## Materilas and methods

### Cell culture, cell lentivirus infection, and cell transfection

HCT116 and RKO cells (ATCC, Manassas, Virginia, USA) were cultured in Dulbecco’s modified Eagle medium (DMEM) (Invitrogen, Carlsbad, CA) containing 10% fetal bovine serum (FBS) (Invitrogen) at 37 °C in a humidified atmosphere of 5% CO_2_.

Lentivirus vector carrying human 4IgB7-H3 cDNA was generated by Genechem Co., Ltd (Shanghai, China). An empty backbone vector was used as a control. For lentivirus infection, HCT116 cells or RKO cells were grown to 30% confluence in 6-well plates and were infected with lentiviral particles at a multiplicity of infection (MOI) of 20. The infection efficiency was confirmed by counting GFP-expressing cells under fluorescence microscopy 72 h after infection.

Human B7-H3 siRNA-1 (5′-GCUGUCUGUCUGUCUCAUUTT-3′), human B7-H3 siRNA-2 (5′-GUGCUGGAGAAAGAUCAAATT-3′), human HK2 siRNA (5′-CACGATGAAATTGAACCTGGT-3′) and their control siRNA were purchased from GenePharma Co. Ltd. (Suzhou, China). HCT116 or RKO cells were transfected with siRNA reagents using Lipofectamine 2000 (Invitrogen) according to the manufacturer’s instructions. Transfection efficiency was determined by RT-qPCR and western blot.

### Total RNA isolation and RT-qPCR assays

Total RNA from cultured cells was prepared using TRIzol reagent (Invitrogen) according to the manufacturer’s instructions. For mRNA detection, RT-qPCR assays were performed on a CFX96 Touch^TM^ Real-Time PCR system (Bio-Rad, CA, USA) using EvaGreen Dye (Biotium, Hayward, CA, USA). For analysis of individual genes, 0.5 μg of total RNA was used for DNA synthesis and RT-qPCR was conducted using a SYBR PrimeScript RT-qPCR Kit (Takara Bio, Shiga, Japan). The PCR conditions were as follows: 95 °C for 5 min, and then 40 cycles of amplification for 30 s at 95 °C, 45 s at 60 °C and 45 s at 72 °C. Individual gene expression was normalized to β-actin mRNA. The primer sequences for RT-qPCR of genes are provided in Supplemental Table 1.

### Protein extraction and western blot analysis

HCT116 or RKO cells in 6-well plates were lysed with RIPA Lysis Buffer (Thermo Scientific, Waltham, MA, USA) containing protease inhibitor cocktail (Sigma, St Louis, Missouri, USA) according to the manufacturer’s instructions. Protein concentrations were measured with a Pierce BCA protein assay kit (ThermoScientific, New York, USA). Equal amounts of protein were separated by 10% sodium dodecyl sulfate-polyacrylamide gel electrophoresis (SDS-PAGE) and transferred to a polyvinylidene fluoride (PVDF) membrane (Merck Millipore, Darmstadt, Germany). The antibodies for the western blot analysis in this study were as follows: goat anti-human 4IgB7-H3 (R&D Systems, #AF1027), mouse anti-human HK2 (Novus Biologicals, #NBP1-51643), rabbit anti-human/mouse STAT3 (CST, #12640, MA, USA), rabbit anti-human/mouse Phospho-STAT3 (pSTAT3) (CST, #9145), rabbit anti-human Bcl-2 (abcam, #ab32124), rabbit anti-human/mouse Bax (abcam, #ab32503) and mouse anti-human/mouse β-actin (CST, #3700). The membranes were then developed with Clarity Western ECL substrate (Bio-Rad) and visualized with a ChemiDoc^TM^ MP Imaging System (Bio-Rad).

### Proliferation assay and colony formation assay

Cell viability was assessed using the Cell Counting Kit-8 (CCK8) assay (Dojindo Laboratory, Japan) according to the manufacturer’s protocol. For the L-OHP and 5-FU viability assays, 5000 cells/well were seeded in 96-well plates, and then L-OHP (5 μmol/L) or 5-FU (10 μmol/L) was added (Sigma). Viability was measured after treatment with L-OHP or 5-FU for 48 h.

For the colony formation assay, cells were seeded in 12-well plates and then treated with 5 μmol/L L-OHP. After 2 h, the cells were harvested using trypsin, collected, and reseeded at a density of 2000 cells/well in 12-well plates. Then, the colonies were fixed and stained with 0.1% crystal violet (Sigma) 14 days later.

### Cell apoptosis assay

Cell apoptosis was measured using an Annexin-V-PE/7-AAD double staining apoptosis kit (BD, Franklin Lakes, NJ) according to the manufacturer’s instructions. Cells were analyzed by flow cytometry (Beckman Coulter, CA, USA). Annexin-V+/7-AAD- cells and Annexin-V+/7-AAD+cells were the apoptotic cells.

### Glucose consumption and lactate production assay

Glucose consumption and lactate production were tested using a Glucose Assay kit (Sigma) and a Lactate Assay kit (Sigma), respectively, as described previously, respectively^50^. The values were normalized to total protein concentration (Pierce BCA protein assay kit, ThermoScientific).

### Xenograft tumor

All manipulations involving live mice were performed in accordance with currently prescribed guidelines and under a protocol approved by the Institutional Animal Care and Use Committee at Soochow University (Suzhou, China). Six–eight-weeks-old female BALB/c athymic nude mice were obtained from the Experimental Animal Center of the Chinese Academy of Medicine Sciences of Soochow University and maintained in a specific pathogen-free environment. For xenograft experiments, B7-H3-overexpressing HCT116 cells (5 × 10^6^) were harvested, resuspended in 150 μl of phosphate buffered solution (PBS) (Beyotime, Shanghai, China) and injected subcutaneously into the right flank of each mouse. Tumor volume (mean ± sem; mm^3^) was calculated according to the following equation: *V* (mm^3^) = S^2^(mm^2^) × L(mm)/2, where S and L are the smallest and the largest perpendicular tumor diameters, respectively^51^.

To determine the resistant effect of B7-H3 on L-OHP in vivo, the mice were randomly divided into the following groups: empty vector-HCT116 (HCT116-EV), B7-H3-overexpression HCT116 (HCT116-B7-H3), HCT116-EV-L-OHP, and HCT116-B7-H3-L-OHP. The mice in the HCT116-EV-L-OHP and HCT116-B7-H3-L-OHP groups were intraperitoneally injected with L-OHP (5 mg/kg) twice a week for 2 weeks. The mice in the HCT116-EV and HCT116-B7-H3 groups received vehicle control. L-OHP treatment began on day 6. To investigate whether the resistant effect of B7-H3 was based on HK2 or aerobic glycolysis, 500 mg/kg 2-DG (Sigma) or vehicle control was given intraperitoneally into mice in the HCT116-B7-H3 group mice one day before of intraperitoneal injection of L-OHP (5 mg/kg). L-OHP treatment began on day 6.

### Tissue specimens

Paraffin blocks of 126 CRC specimens from patients were obtained from the First Affiliated Hospital of Soochow University (Suzhou, China); the specimens contained CRC tissues and adjacent normal tissues and were identified as CRC under hematoxylin and eosin (H&E) staining. Detailed clinicopathological information is provided in Supplemental Table 2. Ethics approval was obtained from the Institutional Review Board of Soochow University. Informed consent was also obtained from patients for experimentation.

### Immunohistochemistry

Immunohistochemistry (IHC) was performed as previously described^6^. Briefly, sections from paraffin embedded tissues were incubated with goat anti-human 4IgB7-H3 antibody (R&D Systems, #AF1027) or mouse anti-human HK2 antibody (Novus Biologicals, #NBP1-51643) overnight at 4 °C. This step was followed by staining (45 min at room temperature) with the corresponding HRP-labeled rabbit anti-goat secondary antibody or goat anti-mouse secondary antibody (Invitrogen). Next, the sections were visualized by staining with 3,3′-diaminobenzidine (Biocare Medical, California, USA) and counterstaining with hematoxylin (Sigma).

All sections were then reviewed blindly by two experienced pathologists (Dr. Cao and Dr. Zhan). The scoring criteria for B7-H3 and HK2 immunostaining were based on clinical data and adopting the semiquantitative immunoreactive score (IRS) system^52^. Briefly, category A (intensity of immunostaining) was scored using the following criteria: 0, negative; 1, weak; 2, moderate; 3, strong. Category B (percentage of immunoreactive cells) was scored using the following criteria: 1, (0–25%); 2, (26–50%); 3, (51–75%); and 4, (76–100%). Final scores were calculated by multiplying the scores of categories A and B in the same section; the scores ranged from 0 to 12.

### Statistical analysis

The results are expressed as the mean ± standard error (mean ± SEM). The differences between groups were analyzed by two-tailed unpaired Student’s *t* test. In addition, a paired Student’s *t* test was used to analyze the replicated experiments. A value of *P* < 0.05 was considered significant.

## Supplementary information

Supplemental figure legends

Figure S1

Figure S2

Supplemental tables
