# Supplementary material for: B7-H3 promotes aerobic glycolysis and chemoresistance in colorectal cancer cells by regulating HK2
Source: Cell Death Dis. 2019 Apr 5;10(4):308. doi: 10.1038/s41419-019-1549-6 (PMC6450969; doi:10.1038/s41419-019-1549-6)
Supplement: Supplementary file 1 — Supplemental figure legends [file 41419_2019_1549_MOESM1_ESM.docx]

**Supplemental Figure** **Legends**

**Figure S1. B7-H3-overexpressing HCT116 or RKO cells apoptosis after L-OHP treatment was measured by flow cytometry based on Annexin V/7-AAD double staining.** Values are expressed as means (SEMs). Five samples were analyzed per condition, and the experiments were performed in triplicate.

**Figure S2. The level** **HIF-1α were no change in B7H3 expressing HCT116 or RKO cells** **following CoCl_2_ treatment.** The protein level of HIF-1α in overexpressing EV or B7H3 HCT116 or RKO cells following CoCl_2_ treatment. were analysed by Western Blot. β-actin served as loading control. The experiments were performed in triplicate.
