# Supplementary figures and images for: B7-H3 promotes aerobic glycolysis and chemoresistance in colorectal cancer cells by regulating HK2
Source: Cell Death Dis. 2019 Apr 5;10(4):308. doi: 10.1038/s41419-019-1549-6 (PMC6450969; doi:10.1038/s41419-019-1549-6)

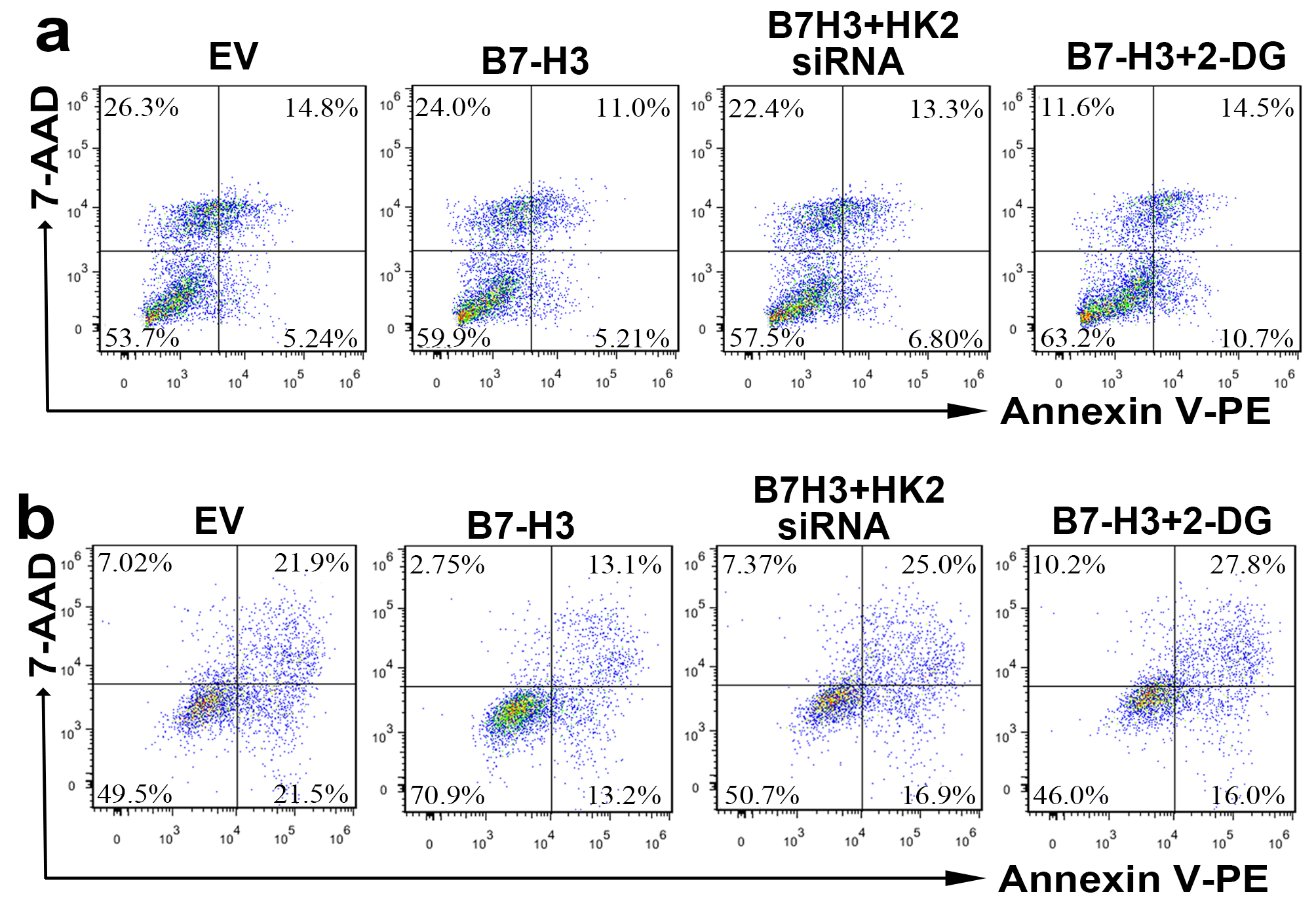

Supplement: Supplementary file 2 — Figure S1 [file 41419_2019_1549_MOESM2_ESM.tif]

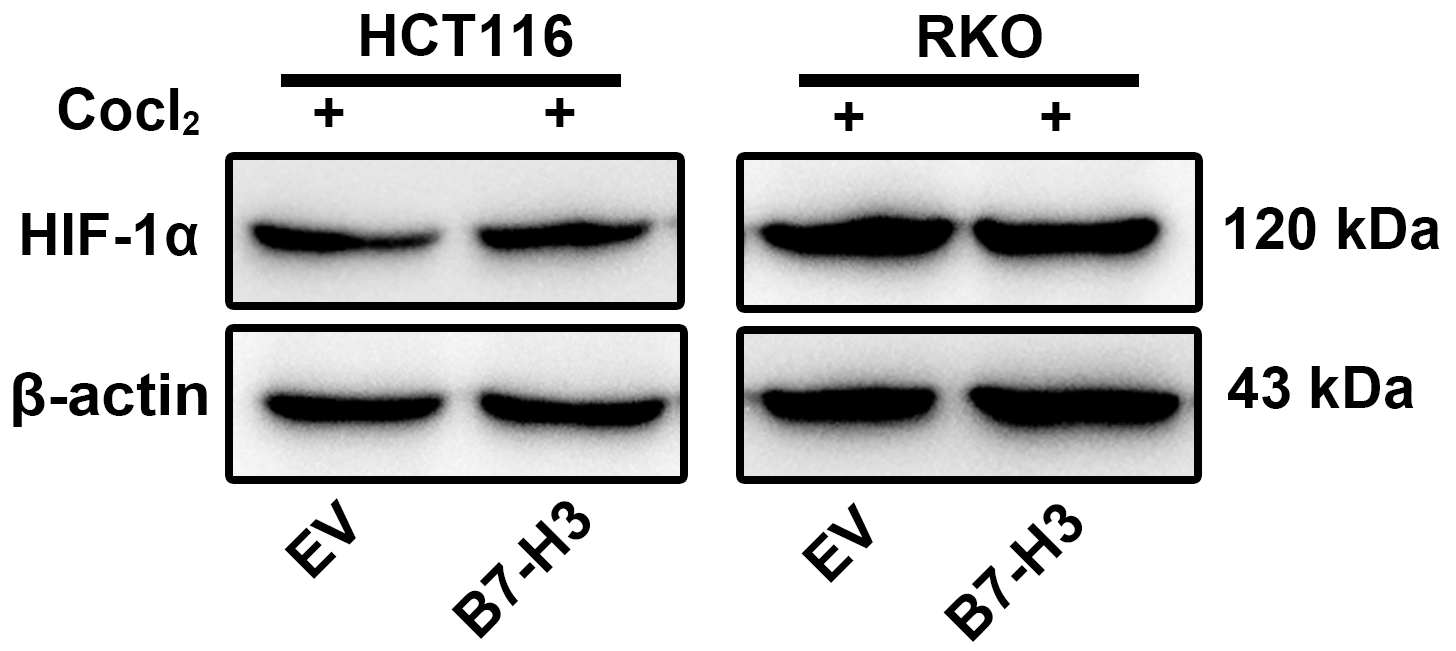

Supplement: Supplementary file 3 — Figure S2 [file 41419_2019_1549_MOESM3_ESM.tif]
