# Supplementary material for: B7-H3 promotes aerobic glycolysis and chemoresistance in colorectal cancer cells by regulating HK2
Source: Cell Death Dis. 2019 Apr 5;10(4):308. doi: 10.1038/s41419-019-1549-6 (PMC6450969; doi:10.1038/s41419-019-1549-6)
Supplement: Supplementary file 4 — Supplemental tables [file 41419_2019_1549_MOESM4_ESM.docx]

**Supplemental tables**

**Supplemental Table 1. Clinical characteristics of patients**

| CRC patients | Number |
| --- | --- |
| NO. of patients | 126 |
| Gender |  |
| Male | 73 |
| Female | 53 |
| Age (years) |  |
| Mean | 60.37 |
| Range | 26-81 |
| Tumor location |  |
| Colon | 73 |
| Rectun | 53 |
| TNM stage |  |
| I-II | 65 |
| III-IV | 61 |

**Supplemental Table 2. primers for qRT-PCR assay of gene**

| Primer Name | Primer Sequence(5’-3’） |
| --- | --- |
| Homo-GLUT1 Forward | GGCCAAGAGTGTGCTAAAGAA |
| Homo-GLUT1 Reverse | ACAGCGTTGATGCCAGACAG |
| Homo-GLUT4 Forward | TGGGCGGCATGATTTCCTC |
| Homo-GLUT4 Reverse | GCCAGGACATTGTTGACCAG |
| Homo-LDHA Forward | ATGGCAACTCTAAAGGATCAGC |
| Homo-LDHA Reverse | CCAACCCCAACAACTGTAATCT |
| Homo-LDHB Forward | TGGTATGGCGTGTGCTATCAG |
| Homo-LDHB Reverse | TTGGCGGTCACAGAATAATCTTT |
| Homo-HK2 Forward | GAGCCACCACTCACCCTACT |
| Homo-HK2 Reverse | CCAGGCATTCGGCAATGTG |
| Homo-PKM2 Forward | ATGTCGAAGCCCCATAGTGAA |
| Homo-PKM2 Reverse | TGGGTGGTGAATCAATGTCCA |
| Homo-HIF-1α Forward | GAGTGCCTCTACCCATACCCT |
| Homo-HIF-1α Reverse | TCGTAGTCGGGATTGTCAAAGT |
| Homo-PDK1 Forward | CTGTGATACGGATCAGAAACCG |
| Homo-PDK1 Reverse | TCCACCAAACAATAAAGAGTGCT |
| Homo-β-actin Forward | CATGTACGTTGCTATCCAGGC |
| Homo-β-actin Reverse | CTCCTTAATGTCACGCACGAT |
